# Agricultural expansion and mining drive forest loss in Ghana despite declining deforestation rates: Evidence from a national-scale assessment

**DOI:** 10.1371/journal.pone.0357446

**Published:** 2026-09-03

**Authors:** Patrick Addo-Fordjour, Francis Emmanuel Awortwi, Caleb Ezekiel Obodai, Francis Boakye Aboagye, Christine Naa Lamiley France, Nana Serwaa Boakye Kusi-Appiah, Derrick Oppong

**Affiliations:** Department of Theoretical and Applied Biology, College of Science, Kwame Nkrumah University of Science and Technology, Kumasi, Ghana; RLBCAU: Rani Lakshmi Bai Central Agricultural University, INDIA

## Abstract

Reliable estimates of deforestation are essential for understanding the dynamics of forest change in order to guide conservation and land management policies. However, considerable variation exists among published estimates of forest loss in Ghana, creating uncertainty regarding the magnitude and drivers of deforestation. Furthermore, deforestation rates have largely been estimated for individual forest reserves or selected landscapes rather than for the entire country. To address this gap, the present study provided a nationally explicit assessment of deforestation rates and forest conversion pathways across Ghana between 2015 and 2025 using multi-temporal Sentinel-2 imagery and post-classification change detection techniques. Land use land cover maps were generated for 2015, 2020, and 2025 and used to quantify land cover transitions, estimate deforestation rates, and identify the dominant pathways through which forests were converted to other land uses. Our findings showed that the landscape was largely characterised by stable non-forest (73.82%) and stable forest (22.36%) cover over the study period. Nevertheless, Ghana lost approximately 279,227 ha of forest between 2015 and 2025, corresponding to a cumulative deforestation rate of 4.95% and an average annual deforestation rate of 0.40% yr ^−^ ^1^. Forest loss increased from 130,878 ha during 2015–2020 to 149,984 ha during 2020–2025, indicating an acceleration in deforestation. Spatial analyses showed that stable forests were concentrated mainly within the southwestern high forest zone, whereas forest degradation was more widespread across the transition and savanna landscapes. Examination of forest conversion pathways revealed that agricultural expansion and mining were the predominant drivers of deforestation, accounting for 80.4% and 11% of total forest loss, respectively. Over the decade, mined area exhibited the greatest proportional increase (533.9%). The findings indicated that agricultural expansion remains the principal cause of forest loss in Ghana, although the rapid growth of mining activities constitutes an emerging threat to the country’s forest ecosystems. Continued forest loss is likely to undermine carbon sequestration capacity and compromise the contribution of Ghana’s forests to climate-change mitigation. By providing updated and spatially explicit estimates of forest change, this study fills a critical knowledge gap in national-scale deforestation assessment in Ghana. It also provides information relevant to national forest monitoring, land-use planning, and the development of strategies aimed at reducing deforestation and promoting sustainable landscape management.

## Introduction

Forests are among the most important terrestrial ecosystems on Earth, as they provide a wide range of ecological, economic, and social benefits [1]. They serve as major reservoirs of biodiversity, support hydrological regulation, protect soils from erosion, and contribute to climate regulation through carbon sequestration [2]. Furthermore, forests provide essential goods and services that sustain the livelihoods of millions of people worldwide [1]. In many tropical countries, forests contribute directly to food security, energy supply, employment, and rural development through the provision of timber and non-timber forest products [3,4]. Consequently, the conservation and sustainable management of forest ecosystems are key to biodiversity conservation, climate change mitigation, and sustainable development [5]. To this end, knowledge of the human activities that drive forest degradation and loss is essential for informing effective conservation and management practices.

Despite their importance, forests continue to face increasing pressure from anthropogenic activities. Agricultural expansion, logging, mining, infrastructure development, urbanisation, and population growth have been identified as major drivers of forest loss across tropical regions [6–10]. These activities often result in deforestation and forest degradation [11,12] that lead to habitat fragmentation, loss of biodiversity and ecosystem services, reductions in carbon storage, and increased greenhouse gas emissions [13]. Similar trends have been observed in Ghana, where forests have undergone substantial decline over the past several decades due to increasing human pressure [7,14–16]. Consistent with the pattern already described for tropical regions [17], agricultural expansion, particularly tree crop and food crop production, has been identified as the principal driver of forest loss in Ghana [7]. Similarly, as observed across tropical regions, logging and mining continue to exert significant pressure on Ghana’s forest ecosystems [8,10,11,15]. However, despite the increasing pressure on tropical forests from competing land uses, global deforestation rates have shown a declining trend in recent decades [18]. Thus, understanding the magnitude, patterns, and drivers of forest loss is essential for designing effective conservation and land use policies.

Accurate estimation of deforestation rates is essential for evaluating the effectiveness of forest conservation initiatives, monitoring progress towards national and international climate commitments, and informing sustainable land use planning. However, reported deforestation rates in Ghana vary considerably depending on the spatial extent, forest definition, and methodological approach employed. Similar discrepancies between forest assessments have been reported by Masolele et al. [19], who attributed such differences to variations in forest definitions, mapping methodologies, and assessment scales. Indeed, Acheampong et al. [7] reported annual deforestation rates of 1.1% and 2.0% for dense and logged forests, respectively, within forest reserves in the Ashanti Region, Ghana between 1986 and 2015. These estimates were derived from specific forest reserve landscapes, and therefore may not be representative of deforestation dynamics across the country as a whole [15]. Similarly, Dembélé et al. [20] reported annual deforestation rates of 0.64% for the Bobiri Forest Reserve between 1986 and 2022. Asamoah [15] reported a deforestation rate of 1.8% based on analyses of forest areas associated with the operations of 20 timber companies in Ghana. Furthermore, the Forestry Commission reported that Ghana’s deforestation rate occurs at approximately 3.6% per annum since 2001 [16], although the spatial extent and methodological basis of this estimate were not clearly specified. Consequently, robust estimates of deforestation derived from direct LULC change analyses across the entire land area of Ghana remain limited. At the national scale, FAO [21] estimated an average annual forest loss rate of approximately 2.0% between 1990 and 2010 based on changes in total forest area. However, this estimate was derived from the synthesis of national datasets and reporting sources rather than from a direct assessment of LULC transitions. Consequently, it did not identify the specific pathways through which forests are converted to alternative land uses, limiting its usefulness for understanding the proximate drivers of deforestation.

Although previous studies provided valuable information on forest dynamics within specific landscapes, they did not adequately capture land use transitions occurring outside reserve boundaries, where agricultural expansion, settlement development, mining activities, and other land use changes also contribute substantially to forest conversion [22]. Moreover, estimates derived from localised landscapes may either overestimate or underestimate national deforestation rates depending on the intensity of forest loss occurring within the selected study areas. Consequently, comparisons among existing studies are often difficult because observed differences may reflect variation in study extent and methodology rather than actual differences in deforestation dynamics [19,23]. Beyond quantifying the magnitude of forest loss, understanding the land use transitions responsible for deforestation is equally important [19]. Information on the ultimate destinations of deforested land and the relative contributions of different land use classes to forest conversion is essential for identifying dominant deforestation pathways and designing targeted conservation and land management interventions. However, existing studies have rarely combined the quantification of deforestation rates with analyses of the land use transitions responsible for forest loss at the national scale. To the best of our knowledge, no previous study has explicitly quantified deforestation rates from LULC change analyses encompassing the entire land area of Ghana while simultaneously identifying the major land use transitions associated with forest loss. This represents an important knowledge gap because national-scale assessments are necessary for evaluating overall forest loss and land use conversion pathways to inform evidence-based conservation and land management strategies [19,24,25].

Recent advances in Earth observation and geospatial analysis have considerably improved the monitoring of LULC dynamics through the availability of high-resolution satellite imagery, cloud-based processing platforms, improved image analysis techniques, and spatial modelling techniques [26–28]. These developments have enhanced the detection of landscape transformations and provided valuable insights into ecosystem change, habitat connectivity, and sustainable land management across diverse environmental settings [29,30]. These technological advances now provide an opportunity to undertake more comprehensive and spatially explicit assessments of land use dynamics at national scales than was previously possible. Such assessments can improve understanding of interactions among forests, agriculture, settlements, mining, and other land uses, thereby providing stronger evidence to support conservation and sustainable land-use planning.

In view of the foregoing, the present study was undertaken to provide a nationally explicit assessment of deforestation rates and forest conversion pathways across Ghana, thereby contributing to a more comprehensive understanding of forest loss patterns in the country. Specifically, the study quantified national deforestation rates, identified major forest conversion pathways, and evaluated the relative contributions of different land use classes to forest loss. We also examined the role of forest degradation in shaping contemporary forest dynamics in Ghana. By providing nationally representative estimates of forest loss and its underlying conversion pathways, this study contributes important information to support forest conservation, climate change mitigation, and sustainable land use planning in Ghana.

## Methods

### Study area

We conducted a nationwide study of Ghana located between latitudes 4°44′ N and 11°11′ N and longitudes 3°15′ W and 1°12′ E. Ghana has a total area of 238,535 km^2^, which is about 0.8% of African land area. Ghana has several types of forests with rainfall regimes that are either unimodal or bimodal and annual rainfall ranging from 600–1000 mm in the Coastal Scrub and Thicket Forest to over 2200 mm in the Wet Evergreen forest. The bimodal rainfall regime is characteristic of the southern and central forest zones. It comprises two rainy seasons: the major rains, which occur between April and July, and the minor rains, which occur between September and November. In contrast, the unimodal rainfall regime is characteristic of the forest–savanna transition and northern areas. It consists of a single rainy season extending from May to October and a dry season from November to April. The dry season is usually associated with harmattan. Temperatures within these forest zones typically range from 24 °C–27 °C in the Wet Evergreen Forests to 26°C–34°C in the Forests Savanna Transition zone.

The traditional land uses in Ghana are small- and large-scale farming, forestry, wood fuel, cattle grazing, urbanisation, tree plantations of exotic and indigenous species (cocoa, rubber, timber), and game/park reserves. In Ghana, agriculture is practiced both off-reserve and within some forest reserves. Within reserves, farming is permitted under the Taungya system, whereby farmers cultivate food crops alongside tree plantations established under the control and supervision of the Forestry Commission. Timber exploitation takes place within timber contract areas, which cover both on- and off-reserves.

### Field site access and permits

Field verification was conducted across Ghana in both forest reserve and off-reserve areas. Permission to access forest reserves was obtained from the Forest Services Division of the Forestry Commission of Ghana, while observations in off-reserve areas were conducted from publicly accessible locations or with the permission of the relevant landowners or communities. No biological specimens were collected during the study.

### Remote sensing data processing and land use land cover analysis

Multi-temporal satellite imagery was used to assess land use and land cover (LULC) changes in Ghana from 2015 to 2025. Administrative boundary data for Ghana were obtained from the Simplemaps Ghana GIS database and used to produce the maps. The shapefile represents Ghana’s current 16 administrative regions and is licensed under the Creative Commons Attribution 4.0 (CC BY 4.0) license [31]. The satellite images and vector datasets were projected into the Universal Transverse Mercator (UTM) coordinate system (Zone 30N) to ensure spatial consistency and minimise geometric distortions. The imagery was subsequently clipped to the study area boundary prior to analysis. To improve discrimination among land cover classes, the Normalized Difference Vegetation Index (NDVI), Normalized Difference Nitrogen Index (NDNI), Bare Soil Index (BSI), and Normalized Difference Built-up Index (NDBI) were calculated and incorporated into the image composites. These indices enhance the spectral separability of vegetation, bare soil, and built-up areas, thereby improving classification accuracy.

Reference data were generated through visual interpretation of Landsat imagery, Sentinel-2 imagery, high-resolution Google Earth imagery, and existing national land cover maps published by the Forestry Commission. Google Earth imagery was used solely as ancillary reference information for the identification and validation of reference samples and was not used as a basemap or reproduced in any figure. On-screen digitisation of visually distinct land cover features, supported by expert knowledge of the landscape, was used to develop the reference dataset. To ensure that the reference samples were sufficiently represented and spatially well distributed across all land cover classes, a stratified random sampling approach was adopted. Stratification was based on an unsupervised K-means classification, which was used to identify broad spectral groups and guide the distribution of reference samples. The resulting spectral classes were subsequently reclassified into four broad categories (forest, tree crops, non-woody vegetation, and non-vegetation) to facilitate representative sampling.

Land use and land cover classification was performed using the supervised Maximum Likelihood classification algorithm in ENVI 5.3. For each epoch considered in this study (2015, 2020, and 2025), a total of 5,000 and 2,143 ground reference points were used for classifier training and independent validation, respectively. Eight land cover classes were identified for all study years: closed forest, open forest/agroforestry, water, cultivated/grass, settlement/bareland, tree crops, mangrove, and mined area.

### Accuracy assessment and change analysis

The reliability of the classified maps was evaluated using the validation dataset, which comprised 30% of the reference samples withheld during model development. Error matrices generated in ERDAS Imagine 2018 were used to derive producer’s accuracy, user’s accuracy, overall accuracy, and the kappa statistic. These measures were employed to quantify classification performance and determine the consistency of the LULC maps.

Changes in land cover were assessed using a post-classification comparison approach implemented through the Matrix Union function in ERDAS Imagine 2018. Pixel-by-pixel comparisons were performed for the periods 2015–2020, 2020–2025, and 2015–2025 to quantify transitions among LULC classes. Transition matrices were used to quantify the extent and direction of land cover transitions and to elucidate the dominant pathways of forest conversion and deforestation during the periods 2015–2020, 2020–2025, and 2015–2025.

### Quantification of deforestation rates

We estimated the cumulative deforestation rate over the 10-year period using the following equation.


$$\begin{array}{c} Deforestation\;rate\;(\%)=\;\frac{{A_{\text{1}}-A}_{\text{2}}}{A_{\text{1}}}\times \text{100} \end{array}$$

Where:

A_1_ = initial forest area (ha)

A_2_ = final forest area (ha)

The annual deforestation rate was subsequently estimated using the logarithmic equation proposed by Puyravaud [32]:


$$\begin{array}{c} =\;\frac{\text{1}}{t_{\text{2}}{-t}_{\text{1}}}\text{ln}(\frac{A_{\text{2}}}{A_{\text{1}}})\times \text{1}\text{0}\text{0} \end{array}$$

Where:

r = Annual deforestation rate (% yr^−1^)

A_1_ = initial forest area (ha)

A_2_ = final forest area (ha)

t_1_ = initial year

t_2_ = final year

ln = natural logarithm

## Results

### Classification accuracies

Overall classification accuracy was high across the three assessment years. Overall accuracy increased from 82.8% in 2015 to 85.0% and 85.3% in 2020 and 2025, respectively. Correspondingly, the Kappa coefficient increased from 0.77 in 2015 to 0.80 in both 2020 and 2025. Classification accuracy for individual LULC classes was consistently high across the three assessment years, with user and producer accuracies exceeding 70% for most LULC classes (Table 1). Cultivated/grass land exhibited the highest and most stable user accuracy (93.6–94.3%) and producer accuracy (87.6–89.0%). Water bodies and mined area also showed high classification performance, with producer accuracies exceeding 90% in all years. Closed forest and open forest/agroforestry were classified with moderate to high accuracy. The producer accuracies for these LULC classes ranged from 71.6–78.9% and 84.9–88.0%, respectively. Settlement/bare land displayed relatively high user accuracy (88.1–95.8%) and producer accuracy (78.2–85.0%). Tree crops exhibited comparatively lower producer accuracy (67.0–71.7). Mangroves recorded the lowest user accuracy (51.3–55.4%) despite having very high producer accuracy (95.1–100%).

**Table 1:** Accuracy metrics for individual land use land cover (LULC) classes in Ghana for 2015, 2020, and 2025.

LULC	2015 Accuracy		2020 Accuracy		2025 Accuracy	
	User	Producer	User	Producer	User	Producer
Closed Forest	78	71.6	74.1	76.1	75.4	78.9
Open Forest/Agroforestry	68.3	84.9	74.3	88	75.6	88
Water	88.2	94.9	91.4	93.7	88.9	91.1
Cultivated/grass	93.6	87.6	94.2	88.5	94.3	89
Settlement/Bare Land	88.1	78.2	88.1	83.5	95.8	85
Tree crops	79.9	67	84.8	71.7	82	70.8
Mangrove	53.2	100	51.3	95.1	55.4	100
Mined area	83.1	91.4	80	91.4	76.5	92.9

### Land use land cover transition matrix

#### 2015-2020.

**Fig 1:**
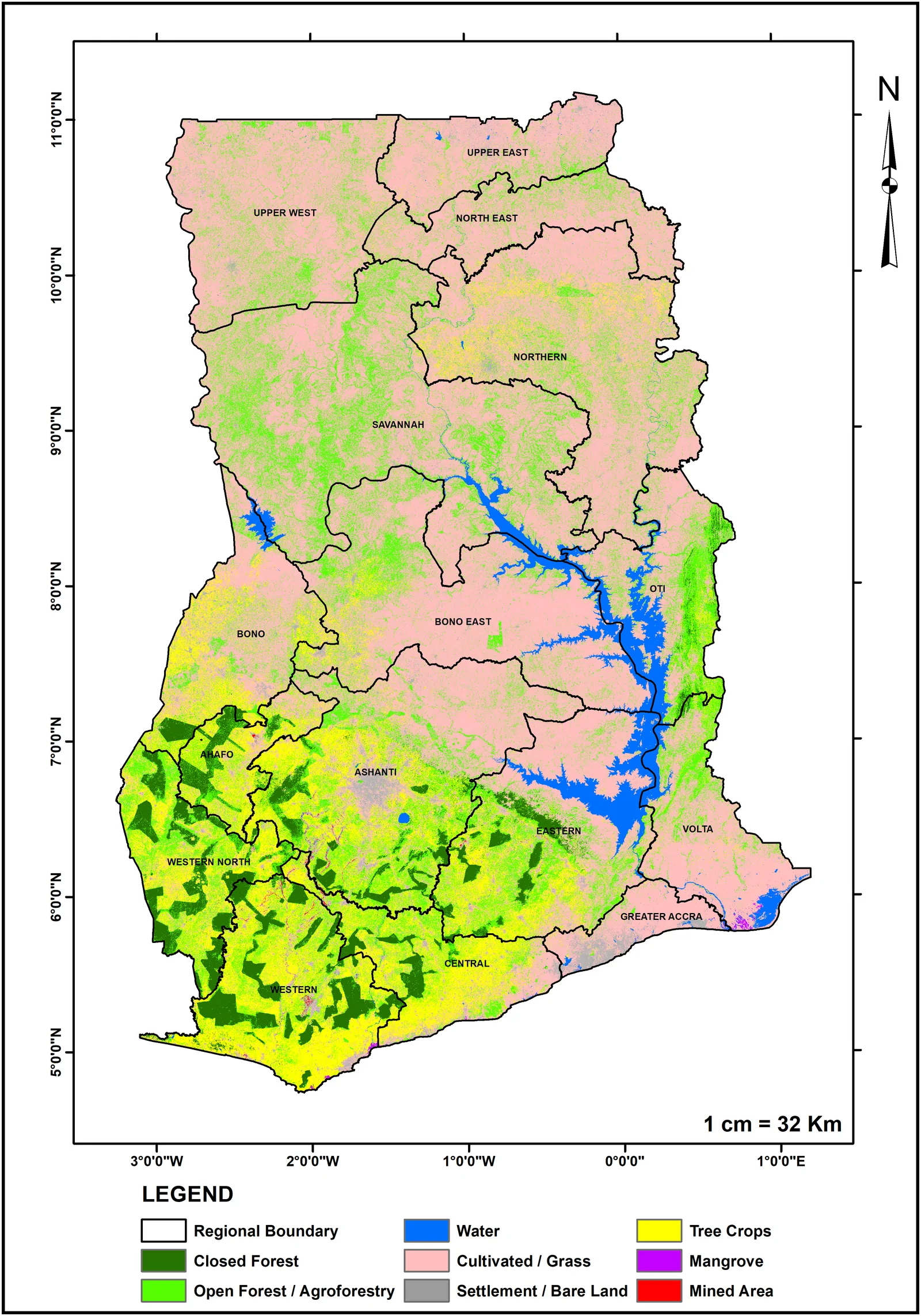
Land use land cover (LULC) maps of Ghana showing the distribution of land cover classes across the national landscape in 2015.

**Fig 2:**
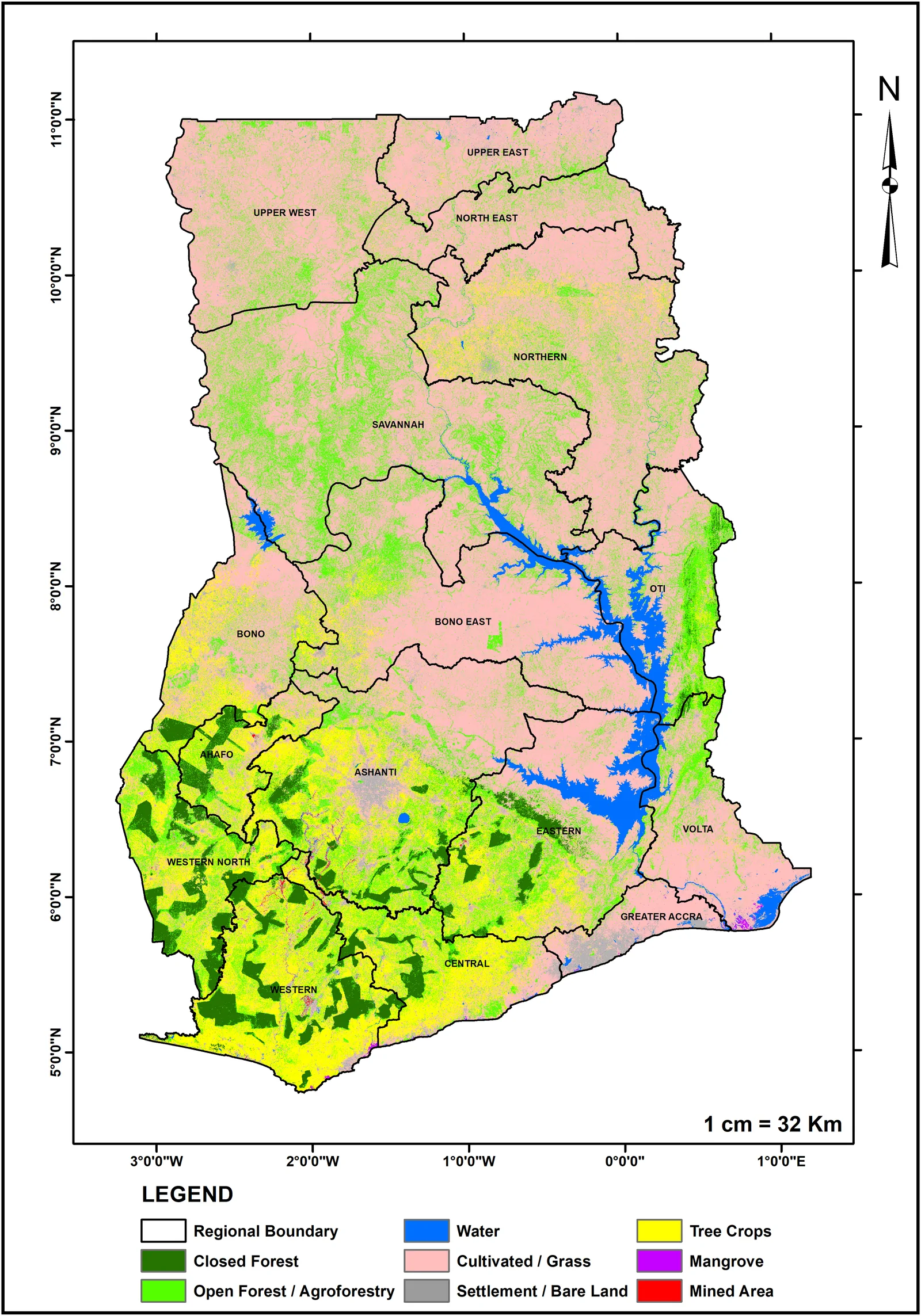
Land use land cover (LULC) maps of Ghana showing the distribution of land cover classes across the national landscape in 2020.

Closed forest cover was relatively stable during the period, with approximately 95.5% remaining intact by 2020 (Table 2; Figs 1 and 2). However, portions of closed forest were converted into tree crops (1.8%), open forest/agroforestry (1.2%), cultivated/grass areas (1.1%) and water bodies (0.2%). Smaller proportions were converted into settlement/bare land and mined area. Open forest/agroforestry was also highly persistent, with only 2.1% transition occurring between 2015 and 2020 (cultivated/grass areas: 1.0%, tree crops: 0.7%, settlement/bare land: 0.28% and mined area: 0.04%, closed forest: 0.04%, water: 0.02%, mangrove: 0.01%).

**Table 2:** Land-use land-cover (LULC) transition matrix (ha) for Ghana between 2015 and 2020. Rows represent the LULC classes in 2015, whereas columns represent the corresponding classes in 2020. Diagonal values indicate areas that remained unchanged, while off-diagonal values represent transitions between classes.

	LULC	2020 (ha)								
		Closed forest	Open forest/ agroforestry	Water	Cultivated/ grass	Settlement/ bareland	Tree crops	Mangrove	Mined area	Total
**2015 (ha)**	**Closed forest**	1,027,362.46	12,679.23	2,492.58	11,983.47	820.74	19,565.80	54.94	778.88	**1,075,738.11**
	**Open forest/** **agroforestry**	1,741.89	4,469,447.19	778.53	45,776.14	12,608.31	33,629.76	427.68	1,666.12	**4,566,075.63**
	**Water**	59.06	746.86	825,862.87	401.03	841.92	412.70	49.36	315.55	**828,689.35**
	**Cultivated/** **grass**	112.20	18,727.99	10,054.82	13,690,257.89	82,478.82	25,938.44	381.39	1,662.01	**13,829,613.58**
	**Settlement/** **bareland**	109.99	325.27	8,256.75	4,577.87	454,376.59	1,270.74	883.99	-	**469,801.20**
	**Tree crops**	403.49	14,655.46	443.34	118,434.18	1,892.90	2,918,603.85	5.03	701.44	**3,055,139.69**
	**Mangrove**	14.25	176.37	61.04	495.78	11.35	209.60	11,828.94	-	**12,797.33**
	**Mined area**	-	0.88	-	61.60	-	21.53	-	15,361.10	**15,445.11**
	**Total**	**1,029,803.33**	**4,516,759.25**	**847,949.93**	**13,871,987.98**	**553,030.63**	**2,999,652.43**	**13,631.33**	**20,485.11**	**23,853,300.00**

Water bodies showed very high stability, with approximately 99.7% remaining unchanged (Table 2). Only negligible proportions transitioned into other land cover classes, indicating limited hydrological change over the study period. Cultivated/grass areas exhibited similarly high persistence, as only 1% of this LULC type was transitioned into settlement/bare land (0.6%), tree crops (0.2%), open forest/agroforestry (0.1%), and others (0.1%). Settlement/bare land showed little change during the study period, with nearly all of the area (96.7%) retaining its original land cover identity. The limited transitions that occurred were directed mainly towards water bodies (1.76%) and cultivated/grass areas (0.97%), while conversions to tree crops, mangroves, open forest/agroforestry and closed forest collectively accounted for less than 1% of the original area.

Land classified as tree crops exhibited limited dynamism during the study period (Table 2). The overwhelming majority persisted in place (95.5%). The most notable change was their conversion to cultivated/grass landscapes, which accounted for 3.9% of the original tree crop extent, while all other transition pathways contributed only marginally to overall change. About 92.4% of mangrove remained unchanged from 2015 to 2020, while small portions (7.6%) were transitioned into other LULC types including cultivated/grass areas (3.9%), tree crops (1.6%) and open forest/agroforestry (1.4%), water (0.48%), closed forest (0.11%), and settlement/bare land (0.09%). Mined area was the most stable anthropogenic land cover class, with 99.5% remaining unchanged by 2020. Only negligible portions transitioned into cultivated/grass areas (0.40%) and tree crops (0.14%), and open forest/agroforestry (0.006%).

#### 2020–2025.

**Fig 3:**
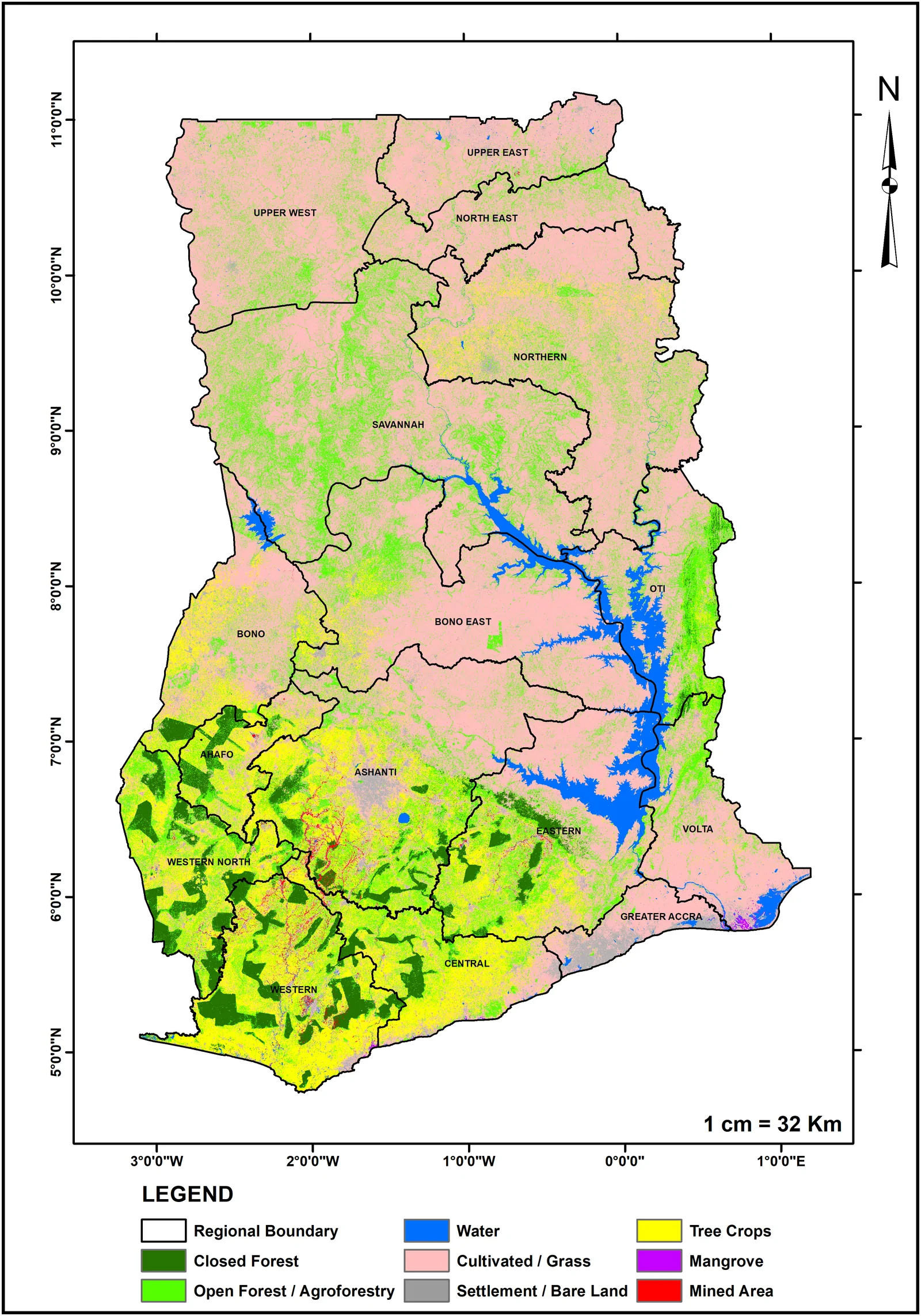
Land use land cover (LULC) maps of Ghana showing the distribution of land cover classes across the national landscape in 2025.

Closed forests still remained relatively stable during the period, with a similar proportion (95.5%) remaining intact by 2025 in relation to 2020 (Table 3; Figs 2 and 3). However, portions transitioned mainly into open forest/agroforestry (2.65%), cultivated/grass areas (1.19%) and mined area (0.62%), settlement/bare areas (0.08%), and others (0.004%). Open forest/agroforestry continued to show high persistence (97.1%), with the remaining proportion being transitioned into cultivated/grass areas (1.44%), tree crops (0.63%), mined area (0.45%), settlement/bare land (0.28%), closed forest (0.07%), and others (0.01%).

**Table 3:** Land-use land-cover (LULC) transition matrix (ha) for Ghana between 2020 and 2025. Rows represent the LULC classes in 2020, whereas columns represent the corresponding classes in 2025. Diagonal values indicate areas that remained unchanged, while off-diagonal values represent transitions between classes.

	LULC	2025 (ha)								
		Closed forest	Open forest/ agroforestry	Water	Cultivated/ grass	Settlement/ bareland	Tree crops	Mangrove	Mined area	Total
**2020 (ha)**	**Closed forest**	983,080.33	27,246.07	-	12,230.75	774.10	0.96	35.96	6,435.16	**1,029,803.33**
	**Open forest/** **agroforestry**	3,099.49	4,386,752.16	307.78	65,153.92	12,712.93	28,362.77	0.03	20,370.17	**4,516,759.25**
	**Water**	0.03	6.29	839,051.01	1,523.99	2,184.49	89.27	0.12	5,094.74	**847,949.93**
	**Cultivated/** **grass**	41.54	6,991.78	24,663.81	13,745,876.08	63,256.35	2,885.21	2,965.66	25,307.54	**13,871,987.98**
	**Settlement/** **bareland**	0.15	3.09	20,238.79	24,375.05	507,150.14	625.13	638.27	-	**553,030.63**
	**Tree crops**	1.66	7,369.21	11,468.00	89,389.84	15,001.11	2,854,931.83	292.17	21,198.62	**2,999,652.43**
	**Mangrove**	120.07	779.18	461.03	2,148.84	77.72	947.86	9,096.41	0.22	**13,631.33**
	**Mined area**	-	-	-	981.46	-	-	-	19,503.65	**20,485.11**
	**Total**	**986,343.27**	**4,429,147.78**	**896,190.43**	**13,941,679.94**	**601,156.83**	**2,887,843.03**	**13,028.63**	**97,910.10**	**23,853,300.00**

Water bodies remained highly stable, with approximately 99% remaining unchanged between 2020 and 2025 (Table 3). This LULC class was mainly transitioned into mined area (0.60%), settlement/bare land (0.25%), and cultivated/grass areas (0.18%). Similarly, cultivated/grass areas exhibited very high stability (99.1%) over the period. However, portions mainly transitioned into settlement/bare land (0.46%), mined area (0.18%) and water bodies (0.18%). By the end of 2025, the greater part of settlement/bare land areas (91.7%) remained unchanged. However, approximately 4.41% transitioned into cultivated/grass areas, while 3.66% converted into water bodies. Smaller proportions were converted into the other LULC classes except mined site.

Like the other LULC classes, tree crops remained relatively stable (95.18%), with the rest being transitioned into the other LULC types: cultivated/grass (2.98%), mined area (0.71%), settlement/bare land (0.50%), water (0.38%), open forest/agroforestry (0.25%) (Table 3). There were smaller conversions into mangrove and closed forest (0.01%). Mangroves exhibited the greatest conversions (33.27%) from 2020 to 2025, resulting in approximately 66.73% of intact mangroves. Significant portions transitioned into cultivated/grass areas (15.76%) and tree crops (6.95%), open forest/agroforestry (5.72%), and water (3.38%). Additionally, there were transitions of smaller portions into closed forest (0.88%), settlement/bare area (0.57%), and mined area (0.002%). Mined area largely remained intact (95.21%) by 2025. Nevertheless, portions transitioned only into cultivated/grass areas (4.79%)

#### 2015–2025.

Closed forest maintained relatively high stability (91.25% intact) between 2015 and 2025 (Table 4; Figs 1 and 3). The other portions were lost to open forest/agroforestry (3.49%), cultivated/grass areas (2.36%) and tree crops (1.76%). Conversion of closed forest to mined area represented 0.77% of the original closed forest extent. There were smaller transitions into water, settlement/bare land, and mangroves, which collectively constituted 0.36% of the original closed forest. Additional closed forest areas emerged mainly from open forest/agroforestry and, to a much lesser extent, from mangrove, tree crops, cultivated/grass land, water, and settlement/bare land. Overall, closed forest declined from 1,075,738.11 ha in 2015–986,343.27 ha in 2025, representing a decrease of 8.3%.

**Table 4:** Land-use land-cover (LULC) transition matrix (ha) for Ghana between 2015 and 2025. Rows represent the LULC classes in 2015, whereas columns represent the corresponding classes in 2025. Diagonal values indicate areas that remained unchanged, while off-diagonal values represent transitions between classes.

		2025 (ha)								
	LULC	Closed forest	Open forest/ agroforestry	Water	Cultivated/ grass	Settlement/ bareland	Tree crops	Mangrove	Mined area	Total
**2015 (ha)**	**Closed forest**	981,659.74	37,632.74	2,226.07	25,373.16	1,586.12	18,908.36	66.97	8,284.95	**1,075,738.11**
	**Open forest/** **agroforestry**	4,042.87	4,342,843.77	2,248.26	116,409.72	17,374.18	60,462.23	263.08	22,431.52	**4,566,075.63**
	**Water**	43.53	674.43	821,096.91	1,324.92	2,249.23	317.85	80.84	2,901.66	**828,689.35**
	**Cultivated/** **grass**	132.85	25,002.68	33,263.67	13,572,048.25	142,081.43	27,684.44	3,187.51	26,212.75	**13,829,613.58**
	**Settlement/** **bareland**	73.36	424.71	25,106.55	18,596.02	421,656.52	1,637.69	1,347.04	959.32	**469,801.20**
	**Tree crops**	260.79	21,622.20	11,714.88	205,262.83	16,036.42	2,777,688.10	285.48	22,269.00	**3,055,139.69**
	**Mangrove**	130.13	947.25	534.10	2,068.15	172.93	1,144.37	7,797.71	2.68	**12,797.33**
	**Mined area**	-	-	-	596.89	-	-	-	14,848.22	**15,445.11**
	**Total**	**986,343.27**	**4,429,147.78**	**896,190.43**	**13,941,679.94**	**601,156.83**	**2,887,843.03**	**13,028.63**	**97,910.10**	**23,853,300.00**

We recorded major conversions of open forest/agroforestry into cultivated/grass (2.55%), tree crops (1.32%), mined area (0.49%), and settlement/bare land (0.38%) (Table 4). Minor transitions of the open forest/agroforestry included closed forest (0.09%), water (0.05%), and mangroves (0.01%). By the end of 2025, 95.11% of the open forest/agroforestry remained untouched. At the same time, gains originated primarily from closed forest, cultivated/grass land, tree crops, and mangrove. Nevertheless, total open forest/agroforestry cover declined from 4,566,075.63 ha in 2015–4,429,147.78 ha in 2025, representing a decrease of 3.0%.

Water bodies largely remained intact (99.1%), with only a small proportion (0.90%) transitioning into other LULC types (Table 4). Mined area accounted for the greatest portion of the original water body loss (0.35%), followed by settlement/bare land (0.27%). Smaller proportions were transitioned into cultivated/grass areas, open forest/agroforestry, tree crops, mangroves, and closed forest. Additional water bodies emerged mainly from cultivated/grass land and settlement/bare land, with smaller contributions from tree crops, mangrove, and forested areas. Overall, water extent increased by 8.1% (828,689.35 ha to 896,190.43 ha) during the period. During 2015–2025, cultivated/grass land cover lost a small portion by conversion into settlement/bare land (1.02%), water (0.24%), tree crops (0.20%), mined area (0.19%), open forest/agroforestry (0.18%), mangrove (0.02%), and closed forest (0.001%). Consequently, most of the original cultivated/grass land (98.2%) persisted during the period. Furthermore, substantial gains occurred from open forest/agroforestry, tree crops, closed forest, settlement/bare land, and mangrove. Consequently, cultivated/grass land increased from 13,829,613.58 ha in 2015–13,941,679.94 ha in 2025, representing an increase of 0.8%.

Settlement/bare land areas were comparatively less stable, with only 89.8% remaining unchanged by 2025 (Table 4). The original settlement/bare land areas were converted primarily into water (5.34%) and cultivated/grass (3.96%). There were transitions into other LULC types that constituted about 0.95% of settlement/bare land areas. In spite of these losses, additional settlement/bare land areas arose primarily from cultivated/grass land, open forest/agroforestry, tree crops, and water bodies. As a result, settlement/bare land expanded by 28% (469,801.20 ha to 601,156.83 ha). Approximately 90.92% of the tree crop area remained unchanged between 2015 and 2025. Transitions from tree crops occurred primarily to cultivated/grass land (6.72%), followed by mined area (0.73%) and open forest/agroforestry (0.71%). Smaller proportions were converted to settlement/bare land (0.52%), water (0.38%), mangrove (0.01%), and closed forest (0.001%). Although new tree crop areas emerged mainly from cultivated/grass land, open forest/agroforestry, and closed forest, the total tree crop area declined by 5.5% (3,055,139.69 ha to 2,887,843.03 ha).

Mangrove cover experienced the greatest decline among all LULC classes between 2015 and 2025, with only 60.93% of its original extent remaining unchanged (Table 4). Conversion to cultivated/grass land constituted the largest transition pathway (16.16%), followed by conversion to tree crops (8.94%), open forest/agroforestry (4.17%), and water (4.17%). Smaller proportions were converted to settlement/bare land (1.35%), closed forest (1.02%), and mined area (0.02%). Gains in mangrove cover originated mainly from cultivated/grass land, settlement/bare land, tree crops, open forest/agroforestry, and water, resulting in an increase of 1.8% (12,797.33 ha to 13,028.63 ha) during the period. Mined area was substantially stable over the 10-year period (96.14%), with a small portion being converted only into cultivated/grass (3.86%). Despite the loss, additional mined area emerged mainly from open forest/agroforestry, cultivated/grass areas and tree crop systems. The total mined area increased from 15,445.11 ha in 2015–97,910.10 ha in 2025, representing an increase of 533.9%.

### Deforestation, forest degradation and major land cover transition pathways

**Fig 4:**
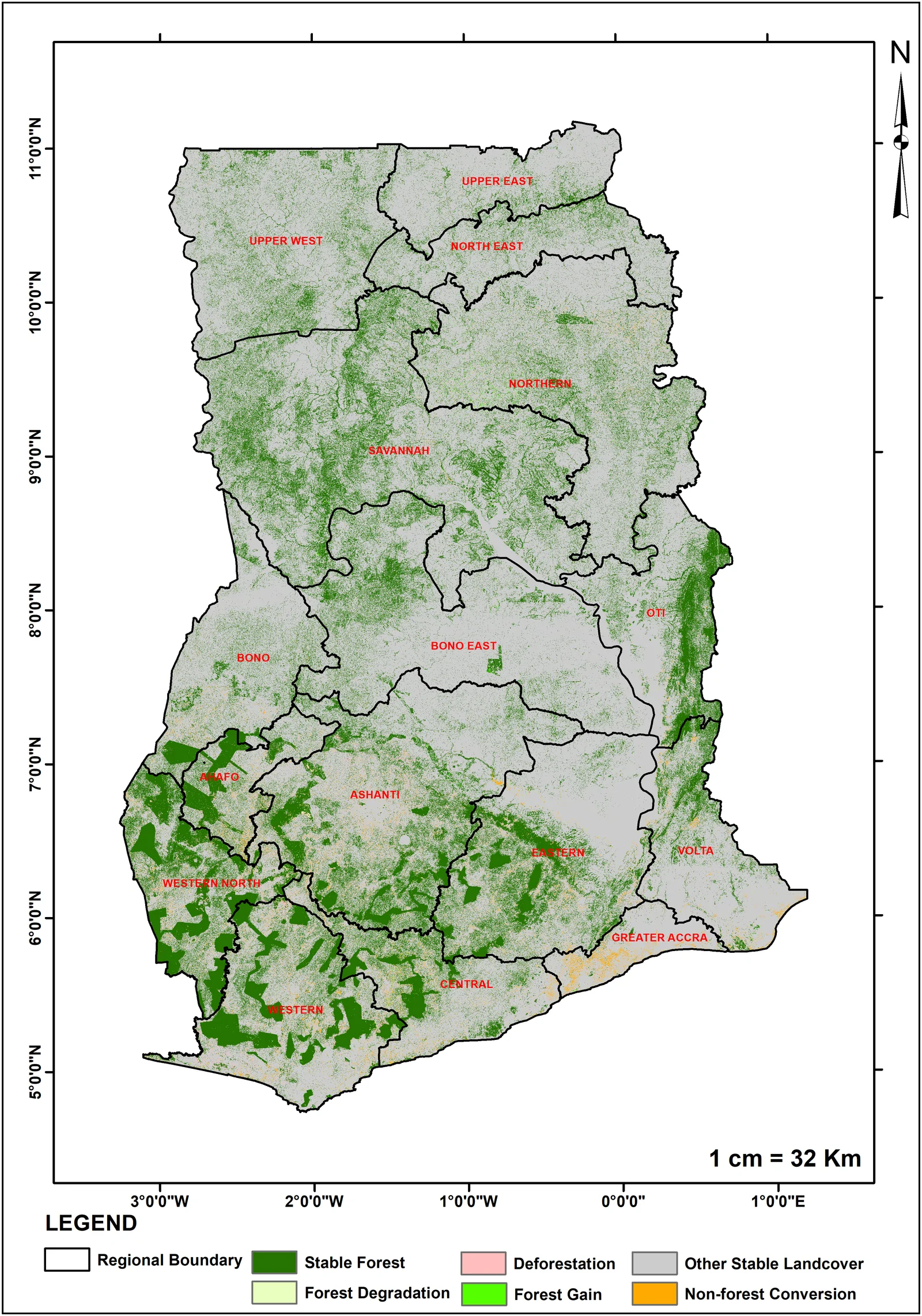
Forest cover change maps of Ghana showing the spatial distribution of stable forests, forest degradation, forest gain, deforestation, non-forest conversion, and other stable land cover classes for 2015–2020.

**Fig 5:**
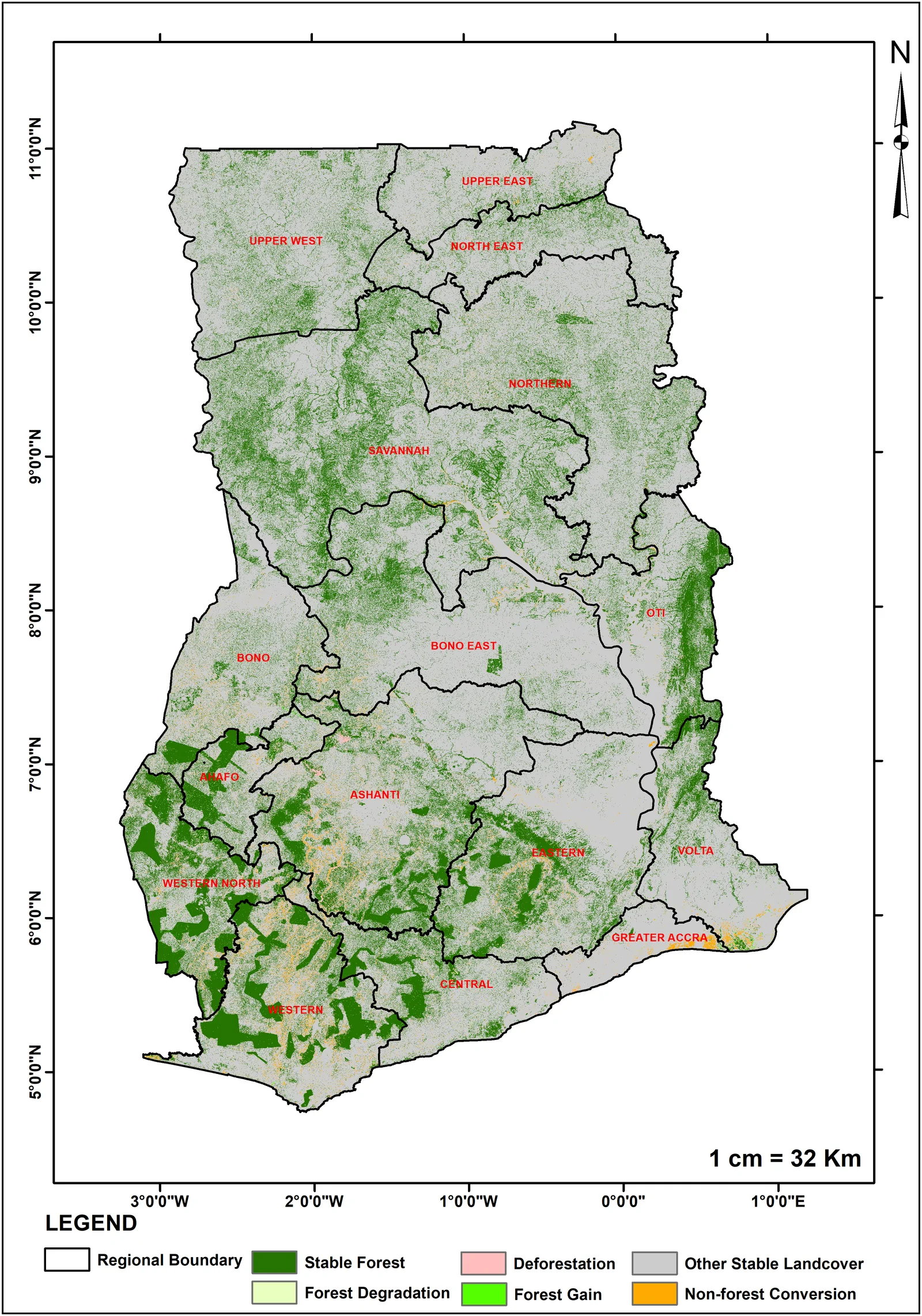
Forest cover change maps of Ghana showing the spatial distribution of stable forests, forest degradation, forest gain, deforestation, non-forest conversion, and other stable land cover classes for 2020–2025.

**Fig 6:**
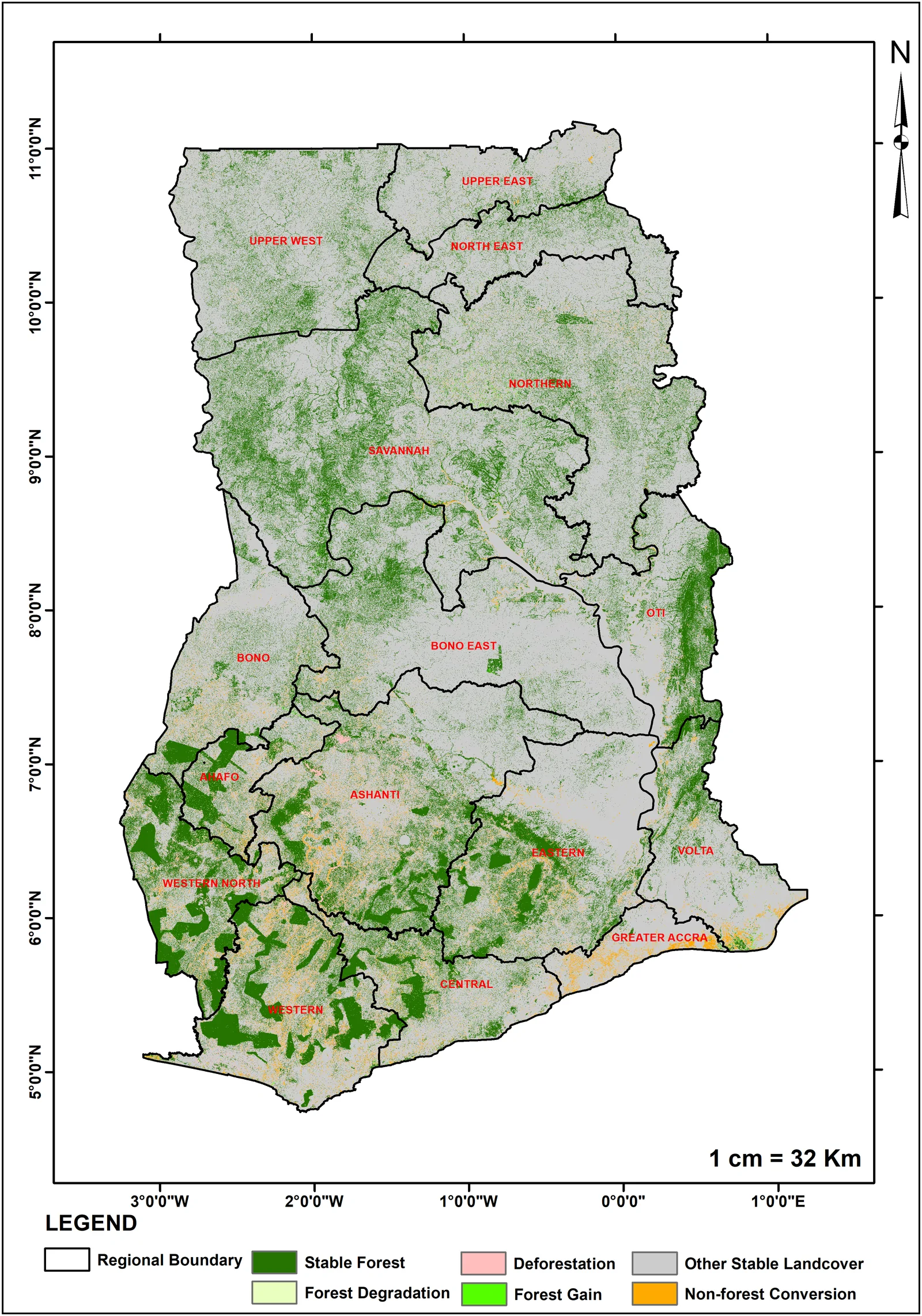
Forest cover change maps of Ghana showing the spatial distribution of stable forests, forest degradation, forest gain, deforestation, non-forest conversion, and other stable land cover classes for 2015–2025.

Ghana lost approximately 130,878.13 ha of forest between 2015 and 2020, corresponding to an annual deforestation rate of 0.34% yr ^−^ ^1^ (overall deforestation rate: 2.32%) (Fig 4). Forest loss increased to 149,984 ha during 2020–2025, with a higher annual deforestation rate of 0.47% yr ^−^ ^1^ (overall deforestation rate: 2.70%) (Fig 5). Overall, the country lost 279,227 ha of forest between 2015 and 2025, amounting to a cumulative deforestation rate of 4.95% and an average annual deforestation rate of 0.40% yr ^−^ ^1^ (Fig 6). The study area was dominated by stable land cover between 2015 and 2025, comprising non-forest (73.82%) and forest (22.36%) classes (Table 4; Fig 6). Forest loss through deforestation accounted for 1.17% of Ghana’s total land area, while a further 0.16% experienced forest degradation. In contrast, forest gain represented 0.24% of the national land area during the study period. Non-forest land cover conversions accounted for 2.26% of Ghana’s total land area. Spatially, stable forests were concentrated mainly in the southwestern high forest zone, whereas forest degradation was widespread across the transition and savanna zones (Figs 4-6).

Analysis of forest conversion pathways revealed that agricultural expansion was the dominant driver of forest loss in Ghana between 2015 and 2025 (Table 5). More than four-fifths (80.4%) of all deforested areas were converted into agricultural land uses, comprising cultivated/grass land and tree-crop systems. Forest conversion to mining accounted for 11.0% of total forest loss, while settlement/bare land and water bodies contributed 6.9% and 1.8%, respectively. Beyond forest conversion, considerable transitions also occurred within agricultural landscapes. Conversion of agricultural land to settlement/bare land constituted the largest agricultural transition pathway, accounting for 62.34% of all agricultural land transitions. A further 19.12% of agricultural land was converted into mined area, whereas 18.54% was transitioned into forest classes.

**Table 5:** Major land cover transition pathways in Ghana between 2015 and 2025.

Transition pathway	Area (ha)
Forest → Agriculture	224,365.99
Forest → Mining	30,719.15
Forest → Settlement/bare land	19,133.24
Forest → Water	5,008.43
Agriculture → Settlement/bare land	158,117.85
Agriculture → Mining	48,481.75
Agriculture → Forest	47,018.52

## Discussion

### Evidence of declining deforestation rates in Ghana

Previous assessments of deforestation in Ghana largely focused on forest reserves, providing valuable information about forest loss within protected and production forest landscapes [7,15]. However, such studies do not capture land cover dynamics occurring outside reserve boundaries, where agricultural expansion, settlement development, and mining activities also contribute substantially to forest conversion [22]. By examining land use and land cover (LULC) transitions across the entire land area of Ghana, the present study provides a more comprehensive national assessment of deforestation and its associated land use trajectories. Although forest reserve studies are essential for evaluating conservation effectiveness within protected forests, national-scale LULC assessments are better suited for quantifying broad-scale deforestation patterns and evaluating the contributions of different land use types to forest loss [19,24,25].

The annual deforestation rate estimated in this study (0.40% yr ^−^ ^1^) was lower than rates reported in several previous assessments of Ghana’s forests (0.64% to 3.6% per annum) depending on the spatial extent, forest definition, and methodological approach employed [7,15,16,19,20]. The lower deforestation rate observed in our study suggests that the rate of forest loss may have slowed at the national scale between 2015 and 2025. Although the factors underlying this trend remain uncertain, the observed decline may coincide with the implementation of several forest conservation initiatives in Ghana, including the Ghana Cocoa Forest REDD+ Programme, landscape restoration efforts, and the promotion of sustainable cocoa agroforestry systems [33,34].

The decline observed in the present study coincided with the implementation of forest conservation initiatives in Ghana and increasing international efforts to address commodity-driven deforestation and promote sustainable supply chains in response to growing concerns about the environmental impacts of forest loss [24,35]. The comparatively lower deforestation rate observed during 2015–2025 is consistent with broader global trends reported over the same period [18]. The FAO reported that global deforestation rates declined during 2015–2025 relative to previous decades, although forests worldwide remained under considerable pressure from agricultural expansion and other anthropogenic activities [18]. This pattern closely mirrors the findings of the present study, where a comparatively lower deforestation rate was accompanied by substantial forest conversion to agricultural land uses and mining areas. This trend indicates that the underlying drivers of forest loss remain active despite a reduction in the overall rate of deforestation. Consequently, the reduction in deforestation rate does not necessarily indicate a reduced pressure on forest ecosystems, as the underlying drivers of forest loss may remain active even when the overall rate of forest conversion slows [18,24].

The comparatively lower deforestation rate observed in the current study may partly reflect differences in the scale of assessment relative to previous studies. Most earlier estimates of deforestation in Ghana were derived from specific forest reserves, timber concession areas, or localised forest landscapes that are often subject to disproportionately high anthropogenic pressure. Consequently, such assessments may overrepresent deforestation dynamics occurring within highly disturbed forest ecosystems. In contrast, the present study adopted a national-scale approach that incorporated all major LULC classes across Ghana, including both areas experiencing forest loss and areas where forest cover remained relatively stable. The broader spatial coverage of the present study may therefore provide a more representative assessment of contemporary forest dynamics at the national level and facilitate the evaluation of major drivers of forest loss across the landscape [24,25]. The lower estimate obtained in the present study suggests that national deforestation trends may be less severe than those inferred from analyses restricted to forest reserves or other high-pressure landscapes. Nevertheless, this does not diminish the ecological significance of forest loss, as more than 279,000 ha of forest were converted to non-forest land uses between 2015 and 2025. Rather, the findings highlight the importance of scale when interpreting deforestation rates, and emphasise the need for national-level assessments to complement localised studies of forest change [7,25].

### Agricultural and mining expansion remain the dominant drivers of deforestation

Despite the apparent reduction in deforestation rates, agriculture remained the dominant proximate driver of forest loss. Agricultural expansion accounted for 80.4% of all deforestation recorded between 2015 and 2025. This finding is consistent with previous studies that identified agricultural expansion as the primary driver of deforestation in Ghana [7,10], Africa [9] and across tropical regions [17,36]. Forest reserve studies have frequently attributed forest loss to agricultural encroachment within reserves [7], whereas national assessments indicate that broader conversion of forests to croplands and agricultural landscapes remains the dominant pathway of deforestation [34]. Given the critical role of agriculture in Ghana’s economy and rural livelihoods [37], it is understandable that agricultural expansion constitutes the major driver of deforestation in the country. In many farming communities, inadequate access to essential inputs such as fertilisers may contribute to declining soil fertility under continuous cultivation. As agricultural productivity declines, farmers may respond by expanding cultivation into new areas, thereby increasing pressure on remaining forest ecosystems [38,39].

Although agricultural land increased by only 0.8% during the study period, approximately 80.4% of the total deforested area was converted to agricultural land. At the same time, substantial areas of agricultural land were converted to settlements/bare land, mining areas, and water bodies. The dominance of forest-to-agriculture transitions may partly reflect displacement effects arising from the conversion of existing agricultural lands to mining areas, settlements/bare land, and water bodies. Previous studies have shown that surface gold mining can substantially reduce the area available for agricultural production and displace farming activities [40]. Such land use competition may compel agricultural expansion into forested areas, thereby creating a feedback mechanism through which non-agricultural land uses indirectly contribute to deforestation.

Another notable finding in our study is that much of the agricultural expansion originated from open forest/agroforestry rather than directly from closed forests. Our findings revealed that the area of open forest/agroforestry converted to agricultural land was approximately four times greater than the area of closed forest converted to agricultural land during the study period. This trend suggests that agricultural expansion increasingly occurred through a gradual process of forest degradation followed by conversion, rather than through direct clearance of intact forests. Such patterns have also been observed in other tropical forests, where degradation preceded complete land use conversion [41–43]. Thus, forest degradation appears to serve as a precursor to deforestation by creating modified forest landscapes that become susceptible to conversion to non-forest land uses.

Our study identified mining as an increasingly important and emerging driver of LULC change in Ghana, showing the largest proportional increase (533.9%) among all the LULC classes over the 10-year period. The rapid expansion of mining observed in our study is consistent with findings from several studies conducted in individual forest reserves across Ghana, which reported the widespread prevalence of artisanal and small-scale gold mining, particularly illegal mining activities (galamsey). These studies identified mining as a major driver of forest loss and degradation in several forested regions of the country where mining concessions and informal mining operations increasingly overlap with agricultural lands and forest reserves. Notably, the period of mining expansion documented in our study coincided with the passage of legislation permitting mining within forest reserves [44]. This policy shift may have facilitated greater access to previously protected forest landscapes and contributed to the observed increase in mining-related LULC change. Consequently, mining activities have resulted in extensive forest clearance, biodiversity loss, and degradation of ecosystem services. Previous studies further reported that the establishment of mining operations was accompanied by an influx of people into mining areas, increasing the demand for land for settlements and agriculture, which exacerbates pressure on surrounding forests and farmlands. The consistency between these localised observations and the nationwide trends observed in our study suggests that mining is becoming a significant contributor to landscape transformation in Ghana.

The current study revealed that agriculture and mining collectively accounted for over 91% of all deforestation between 2015 and 2025, demonstrating that the principal drivers of forest conversion in Ghana remain largely unchanged despite the observed decline in deforestation rates. Furthermore, much of the agricultural expansion occurred through the conversion of open forest and agroforestry systems rather than intact forests, suggesting that forest degradation continues to create pathways for subsequent deforestation. These findings indicate that conservation strategies should focus not only on reducing deforestation rates but also on preventing forest degradation and addressing the underlying land use systems that drive forest conversion.

### Implications for carbon stocks, climate change mitigation, and sustainable land management

The land use transitions observed in the present study have important implications for carbon storage and climate change mitigation in Ghana. Forest ecosystems, particularly intact closed forests, are some of the largest terrestrial carbon reservoirs in the country due to their high biomass and long-term carbon sequestration capacity [45,46]. Consequently, the conversion of forested landscapes to agricultural land, settlements, and mining areas will cause substantial reductions in carbon stocks and carbon sequestration potential of the ecosystems [47,48].

The dominance of agricultural expansion as a major driver of deforestation is particularly significant from a carbon perspective. Given that agricultural systems generally contain considerably lower aboveground biomass than natural forests [49], replacing forests with agricultural land will reduce landscape-level carbon storage. The continued expansion of agriculture therefore presents a major challenge to Ghana’s efforts to reduce greenhouse gas emissions and achieve climate mitigation targets. Forest degradation offers an additional mechanism through which forests lose carbon stock as well as their capacity to sequester and store carbon [48]. Thus, beyond outright deforestation, the substantial conversion of 37,633 ha of closed forest to open forest/agroforestry represents a significant pathway of carbon stock decline in Ghana. While degraded forests may retain some capacity to store carbon, reductions in canopy cover, tree density, and biomass are likely to diminish their overall carbon sequestration potential [45]. The expansion of mining areas has even more serious ramifications for carbon conservation. Unlike agricultural production, mining activities often cause extensive vegetation removal and substantial soil disturbance [50–52]. Such disturbances tend to reduce existing carbon stocks and impair the capacity of affected ecosystems to sequester carbon in the future. Consequently, the conversion of both closed forests and open forest/agroforestry to mined area within the Ghanaian landscape may represent an important source of carbon emissions and long-term ecosystem degradation.

Collectively, our findings suggest that both deforestation and forest degradation are contributing to the erosion of Ghana’s forest carbon stocks. Effective climate change mitigation strategies should therefore extend beyond deforestation prevention to include protection of degraded forests, restoration of disturbed landscapes, sustainable agricultural intensification, and stronger regulation of land use activities that drive forest conversion [53,54]. Such integrated approaches will be essential for maintaining the carbon sequestration function of Ghana’s forests, while simultaneously supporting national development objectives.

The progressive conversion of forested landscapes to agricultural and other human-dominated land uses observed in this study has important implications for landscape-scale ecological management. Continued forest loss and degradation can reduce the capacity of landscapes to sustain essential ecosystem services, including carbon sequestration, water regulation, soil protection, and habitat provision [55]. Consequently, effective land-use planning should integrate biodiversity conservation with sustainable agricultural development, forest restoration, and responsible mining governance to maintain ecosystem resilience and support long-term socio-economic sustainability [56]. These findings are consistent with recent studies that emphasised that incorporating ecosystem service assessments into spatial planning can improve ecological management, maintain soil fertility, and reduce the risks associated with continued land use change [30,57]. Furthermore, the national-scale evidence generated in this study provides a spatially explicit basis for conservation planning and restoration efforts. These findings can also inform sustainable land use policies that balance agriculture, mining, and forest conservation.

## Conclusion

This study provides the first nationally explicit assessment of deforestation rates and forest conversion pathways across the entire land area of Ghana based on multi-temporal Sentinel-2 imagery and land use and land cover change analyses. The findings showed that, although national deforestation rates appear to be declining compared with many previous estimates, forests in Ghana continue to experience substantial pressure from agricultural expansion and the rapid expansion of mining activities. Agricultural expansion remained the dominant driver of forest conversion, whereas the marked increase in mining highlights its growing contribution to ongoing forest loss. The spatially explicit information generated by this study provides an important evidence base for national forest monitoring, conservation planning, sustainable land use management, and climate change mitigation initiatives in Ghana. Future research should integrate higher temporal-resolution satellite imagery with socio-economic and policy data to improve understanding of the interactions among agricultural expansion, mining, policy interventions, and forest dynamics.

Patrick Addo-Fordjour*, Francis Emmanuel Awortwi, Caleb Ezekiel Obodai, Francis Boakye Aboagye, Christine Naa Lamiley France, Nana Serwaa Boakye Kusi-Appiah, Derrick Oppong
